# Tipping the balance in autoimmunity: are regulatory t cells the cause, the cure, or both?

**DOI:** 10.1186/s40348-024-00176-8

**Published:** 2024-03-20

**Authors:** Matthias Hardtke-Wolenski, Sybille Landwehr-Kenzel

**Affiliations:** 1 Hannover Medical School, Department of Gastroenterology Hepatology, Infectious Diseases and Endocrinology, Carl-Neuberg-Str. 1, Hannover, 30625 Germany; 2 University Hospital Essen, Institute of Medical Microbiology, University Duisburg-Essen, Hufelandstraße 55, Essen, 45122 Germany; 3 Hannover Medical School, Department of Pediatric Pneumology, Allergology and Neonatology, Carl-Neuberg-Str. 1, Hannover, 30625 Germany; 4 Hannover Medical School, Institute of Transfusion Medicine and Transplant Engineering, Carl-Neuberg-Str. 1, Hannover, 30625 Germany

**Keywords:** Regulatory T cells, Autoimmunity, Immune dysregulation, Primary Immunodeficiency, Targeted therapy, Cell therapy

## Abstract

Regulatory T cells (Tregs) are a specialized subgroup of T-cell lymphocytes that is crucial for maintaining immune homeostasis and preventing excessive immune responses. Depending on their differentiation route, Tregs can be subdivided into thymically derived Tregs (tTregs) and peripherally induced Tregs (pTregs), which originate from conventional T cells after extrathymic differentiation at peripheral sites. Although the regulatory attributes of tTregs and pTregs partially overlap, their modes of action, protein expression profiles, and functional stability exhibit specific characteristics unique to each subset. Over the last few years, our knowledge of Treg differentiation, maturation, plasticity, and correlations between their phenotypes and functions has increased. Genetic and functional studies in patients with numeric and functional Treg deficiencies have contributed to our mechanistic understanding of immune dysregulation and autoimmune pathologies. This review provides an overview of our current knowledge of Treg biology, discusses monogenetic Treg pathologies and explores the role of Tregs in various other autoimmune disorders. Additionally, we discuss novel approaches that explore Tregs as targets or agents of innovative treatment options.

## Background

The immune system is a complex network of cells and molecules that defends the body against harmful pathogens while maintaining self-tolerance. This delicate balance is orchestrated by various cell types, including T cells, which play a central role in adaptive immunity. Regulatory T cells (Tregs) are a specialized subset of T lymphocytes that play a pivotal role in maintaining immune system homeostasis and preventing excessive immune responses such as autoimmune diseases. More than four decades ago, Tregs emerged as a cornerstone of immunological research. Tregs encompass a heterogeneous population of cells with varying origins and functions. Functionally, Tregs constitute the physiological counterplayers of conventional or cytotoxic T cells and crucially contribute to the maintenance of peripheral immune tolerance [1]. Since their identification by Sakaguchi et al*.* in 1995, regulatory T cells have grown into a large and complex family of regulatory cell populations. Among these, thymically derived Tregs (tTregs), which develop within the thymus before being released into the periphery, represent the majority of peripheral FoxP3 + Tregs. In contrast, peripherally induced Tregs (pTregs) develop from mature conventional FoxP3^−^CD4^+^ T cells upon continuous antigen stimulation in peripheral tissues. During this process, conventional T cells acquire regulatory properties directed by multiple factors, including the presence of certain cytokines and the formation of cellular synapses between various immune cells. This duality highlights the dynamic nature of Treg development and its adaptability to various immunological contexts. Within this review we will summarize biology and functions of Tregs and present the current understand of Tregs deficiencies in monogenetic immunodeficiency and multifactorial autoimmune diseases. Additionally, we will discuss novel therapeutic approaches using Tregs as target or agent to overcome currently unmet medical needs.

### Treg biology and function

The functional characteristics of tTregs and pTregs overlap but differ in terms of their stability. pTregs show a high plasticity and exert regulatory functions only temporarily by transient expression of FoxP3 and additional regulatory elements, which induce the formation of regulatory cytokines [2, 3]. In contrast, tTregs express high levels of FoxP3 [4] and IL2R alpha chain CD25, but low levels of IL-7 receptor CD127. These characteristic elements are critical for the development, function, and homeostasis of tTregs and are tightly linked to their regulatory stability irrespective of the immunologic milieu [5, 6]. However, despite substantial efforts and the discussion of various promising candidates, a phenotypic marker or marker combination that is uniquely expressed by tTregs or allows the discrimination between tTregs and pTregs has not yet been identified [7].

In humans, Tregs constitute only 3–10% of the naïve peripheral CD4^+^ T-cell population. During embryogenesis, Tregs are present within the thymus at 12 gestational weeks and remain stable throughout pregnancy and infancy [8]. Fetal tTregs already express FoxP3 and other markers characteristically linked to their early established immunosuppressive phenotype, *e. g.* the cytotoxic T-lymphocyte-associated protein 4 (CTLA-4) and glucocorticoid-induced TNFR-related protein (GITR) [8, 9]. To protect the human body from autoimmunity, tTregs possess a T-cell receptor (TCR) with a specific affinity for autoantigens [10]. TCR-dependent maturation is mediated by the thymic selection process of tTregs, which focuses on self-protection through the presentation of autoantigens. The presentation of various self-peptides, the so-called tissue-specific antigens (TSA), in medullary thymic epithelial cells (mTECs) is regulated by the transcription factor AIRE (Autoimmune Regulator) and the zinc finger protein Fezf2 [11, 12]. T-cell selection and maturation in a TSA-rich environment ensures immunological self-tolerance. Only T cells bearing TCRs with an intermediate affinity for self-peptides differentiate into tTregs. In contrast, T cells are deleted if they recognize self-peptides with a high-affinity TCR or differentiate into naïve CD4^+^ T cells if self-peptides are recognized with low-affinity TCRs [7, 13–16]. This leads to the effect that pTreg TCRs have a low affinity towards self-antigens but a high affinity for foreign antigens, *for example,* microbial structures. Sequencing analysis of tTregs, pTregs, and conventional T-cell populations revealed that clonal overlap between these populations is particularly low [17–20]. The close link between thymic maturation and stable expression of FoxP3 has been demonstrated to result from a unique pattern of DNA demethylation within an enhancer element of the *FoxP3* promoter region (regulatory T-cell–specific demethylated region [TSDR]) and activation of histone modifications [21–24]. However, even before FoxP3 is functionally expressed, Treg-specific super-enhancers and additional tTreg signature genes, including *CTLA-4, IL2RA,* which encodes the IL-2 receptor CD25, and *IKZF2*, encoding the transcription factor Helios and *IFZF4,* encoding Eos, are activated in Treg progenitor cells [25]. During this process, Satb1, a genome stabilizer, binds to specific genomic sites and supports the opening of chromatin and activation of super-enhancers. Together with the histone lysine methyltransferase MLL4 [26], Satb1 binds to conserved enhancer regions within the FoxP3 promoter, such as CNS0, CNS3, and CNS2, and induces stable activation of these enhancers, as well as the FoxP3 promoter itself [25, 27, 28]. After thymic release, tTregs reside within lymph nodes and peripheral blood. Continuous recognition of self-antigens maintains tTregs in a highly proliferative state and mediates physiological immune homeostasis. Expression of CCR7 and CD62L in naive tTregs and enables Treg homing to the secondary lymphoid organs [29]. There, tTregs—similar to other T-cell subsets—undergo peripheral maturation from naïve (T_N_ CD45RA^+^CCR7^+^) to central memory (T_CM_, CD45RA^−^CCR7^+^), effector memory (*T*_*EM*_, CD45RA^−^CCR7^−^) and finally CD45RA expressing terminally differentiated effector memory cells (T_EMRA_, CD45RA^+^CCR7^−^) [30–34]. Tregs further employ a broad range of chemokine receptors and adhesion molecules for recruitment to inflammatory sites. The release of attractive chemokines at these sites induces Treg migration along a chemotactic gradient. CCR2, CCR4, and CCR5, and particularly CXCR3, CCR6, and CCR8, support recruitment towards sites dominated by Th1, Th2, and Th17 inflammation [35, 36]. The inflammatory response and recruitment of Tregs are further supported by other T-cell subsets and macrophages through the release of IL-2, IL-35, or TGF-β. These not only enhance the recruitment, function, and survival of Tregs, but also support the polarization of naïve CD4^+^ T cells towards pTregs [2]. Conversely, immunosuppressive molecules, including IL-10, IL-35, and TGF-β, are induced in Tregs, which themselves promote crucial survival signals to sustain Tregs in peripheral tissues and mediate non-specific anti-inflammatory signals [37].

**Fig. 1:**
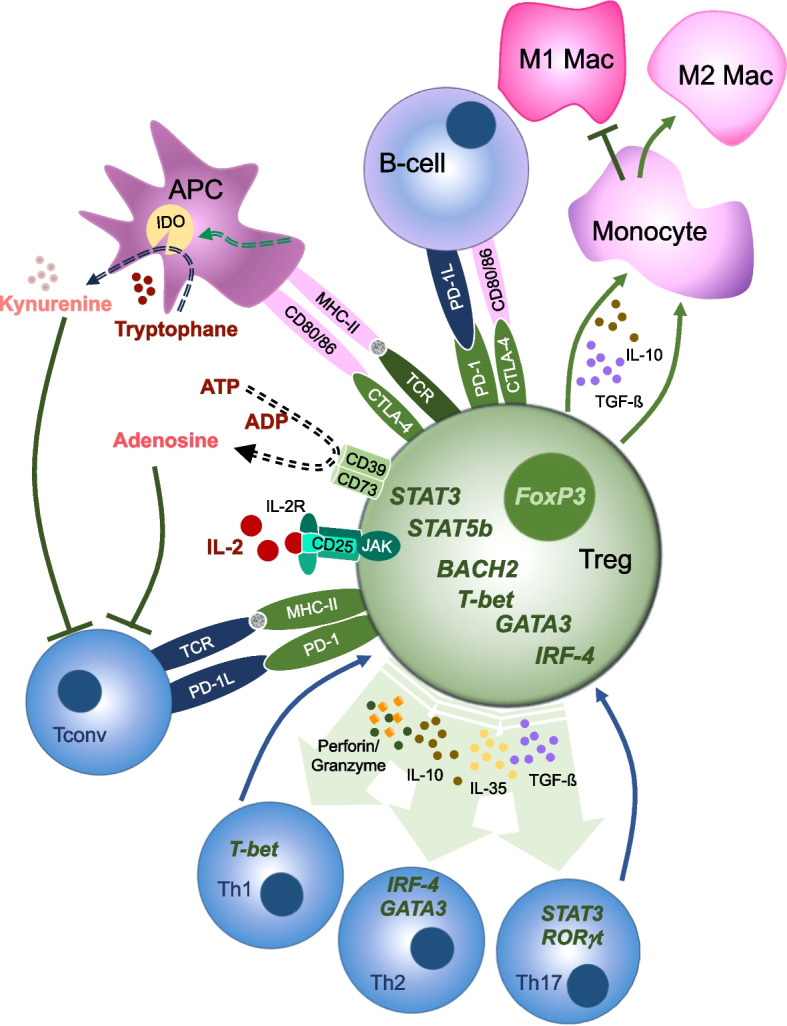
Suppressive Mechanisms of Tregs. Tregs-mediated immune regulation occurs by various cell-contact dependent and contact-independent mechanisms. Anti-inflammatory cytokines, such as TGF-β, IL35, and IL-10, modulate conventional T cells and monocytes towards immunotolerant states. Secretion of Perforin and Granzyme directly targets effector cells and induces apoptosis. By contact-dependent interaction via PD-1/PD-1L, MHC-II/TCR and CTLA4/CD80/CD86 Tregs prevent costimulatory signals in conventional T cells and downregulate the expression of MHC-II and CD80/86 on APCs. Similarly, binding of CTLA-4 to CD80/86 on APCs induces the expression of IDO, converts tryptophan to kynurenine and thereby further suppresses the activation of conventional T cells and promotes regulatory the exertion function in various cell types. Characteristically, high expression of CD25 on Tregs enables Tregs to sequester IL-2 from the environment, thereby limiting IL-2-mediated activation of conventional T cells. Metabolic deprivation is further enhanced by the expression of CD39 on Tregs, which converts ATP to AMP, reducing T-cell proliferation. Tregs may further reduce monocyte differentiation toward pro-inflammatory monocytes and promote M2 macrophage development

Both tTregs and pTregs act as inhibitory immunomodulators through several cell–cell contact-dependent and -independent mechanisms, including inhibition of effector cell proliferation, targeted T-cell cytolysis, ATP consumption (metabolic disruption), and alteration of antigen presentation by macrophages and dendritic cells Fig. 1. Although cytotoxicity is characteristically attributed to conventional CD8^+^ T cells, Tregs have been observed to use Granzyme B and Perforin-mediated cytolysis of target T cells as additional MHC/TCR-independent mechanisms to control inflammation [38–40]. Activation induced expression of CTLA-4 orchestrates broad antigen-specific suppressive functions of Tregs in a contact-dependent fashion [37, 41]. CTLA-4 competitively binds to CD80/CD86 on APCs, including B cells and dendritic cells [37, 42] and thereby reducing the CD28-mediated co-stimulation of conventional T-cells (Tconv). CLTA-4 binding to DCs further reduces the density of antigen-specific MHC-II and CD80/86 on DC via trans-endocytosis [43–46]. Thus, DC lose their capability for MHC-II-mediated antigen-specific activation and T-cell co-stimulation. Antigen presentation on DCs is further reduced by CTLA-4 mediated induction of indoleamine 2,3-dioxygenase (IDO) in DCs. IDO leads to tryptophan depletion and accumulation of the tryptophan metabolite kynurenine, which (i) mediates suppression of proliferation and activation of effector T cells and (ii) promotes differentiation of other immune cells towards a regulatory phenotype [47]. Accordingly, monocytic differentiation is shifted towards anti-inflammatory M2 macrophages, while differentiation to pro-inflammatory M1 macrophages and Th17-cell expansion is inhibited [48–50]. The expression of additional inhibitory surface molecules further contributes to the contact-dependent inhibition of T cells, B-cell and DCs. Among these, PD-1/PD-1L interaction induces phosphorylation of ITSM, which downmodulates intracellular cascades including TCR-, PI3K/AKT-, and Ras/MEK/ERK-signaling and represses T- and B-cell proliferation [51–54]. Additional metabolic pathways used by Tregs not only induce peripheral Treg differentiation and proliferation but also deprive effector T cells of key nutrients, including the degradation of ATP to adenosine by CD39/CD73 and the competitive consumption of key nutrients such as glucose or amino acids [55, 56]. While all T cells express dimeric IL-2 receptors consisting of the γ-chain CD132 and IL2Rβ subunit CD122 with an intermediate IL-2 affinity, Tregs are characterized by the additional and constitutive expression of the high-affinity IL2Rα subunit CD25. This trimeric receptor is characterized by an ca. 1000-fold higher affinity for IL-2. Thus, the expression of CD25 confers a selection advantage during thymic Treg differentiation and induces FoxP3 expression early during Treg development. The phenotypic characteristics of particularly high CD25 expression further correlate with the exertion of regulatory functions. In the periphery, Treg survival and proliferation are strictly dependent on IL-2 produced by activated conventional T cells. Intracellularly, IL-2R signaling in Tregs has recently been shown to be crucially involved in the JAK-STAT5 pathway. Activated transcription factor STAT5 translocates to the nucleus and mediates the induction of CD25 and FoxP3. In addition to the crucial role of CD25 in Treg differentiation and maintenance, extracellular CD25 mediated consumption of IL-2 adds to the key repertoire of Treg mechanisms for suppressing CD8^+^ T-cell proliferation [57]. In contrast, inhibition of CD4^+^ T cells has recently been shown to require IL2R mediated activation of Tregs but to occur independently of extracellular IL-2 deprivation [57]. The situation in pTregs however is different as pTregs may modulate their phenotype and function in response to the microenvironment. In a Th1-mediated milieu, which is characterized by the release of IFN-γ, the expression of the transcription factor T-bet is induced in Tregs. Expression of T-bet (T-box expressed in T cells, also called T-box transcription factor TBX21) promotes the expression of the chemokine receptor CXCR3 and accumulation of Tregs at Th1-cell rich inflammatory sites [58]. In contrast, in a Th-2-rich environment the transcription factor GATA-3 is upregulated and maintains high levels of FoxP3 expression in pTregs [59]. This enables pTregs to contain excessive pro-inflammatory polarization and promote the accumulation of pTregs at Th-2 inflammatory sites [59, 60].. Further, although the expression of the transcription factors IRF-4 [61] and STAT3 [62] is typically assigned to Th-2 and Th-17 cells, respectively, both can be induced in Tregs and enhance their immunomodulatory potential when Th17-cells dominate inflammation [62–64]. In summary, the micromilieu substantially influences how pTregs can modulate their phenotypic and functional phenotype, while tTregs are characterized by an exceptionally high stability.

### Monogenetic treg deficiencies

Numeric or functional Treg deficiencies due to monogenetic variations in the human genome lead to the clinical phenotype of primary immunodeficiency, with predominant signs of polytopic immune dysregulation. Although the clinical phenotype overlaps for various Tregs deficiencies, understanding the underlying molecular defect is important in order to tailor and select appropriate disease-specific therapeutic approaches.

### IPEX-syndrome due to FoxP3 deficiency

The clinical picture of the most profound monogenetic Treg deficiency, named X-linked immune dysregulation, polyendocrinopathy and enteropathy (IPEX) syndrome was first described by Powell et al. in 1982*.* The authors reported a family with 19 affected males from one large family, of whom only two survived the first decade. All affected individuals suffered from severe eczema, enteropathy, thyroiditis, type I diabetes, autoimmune cytopenia and immunodeficiency [65]. Later reports added antibody-mediated intestinal villous atrophy, eosinophilia, high IgE levels, lymphoid hyperplasia, islet-cell hypo- or aplasia, arthritis, kidney involvement, and severe immunodeficiency [66–72]. The *scurfy*mouse model mimics X-linked disease with a clinical phenotype similar to that of patients with IPEX, such as scaly skin, runting, diarrhea, lymphadenopathy, hepatosplenomegaly, and progressive anemia. Even before the molecular cause of IPEX syndrome was identified, this mouse line served as a helpful model for the pathophysiological understanding of IPEX syndrome [73–75]. At the cellular level, CD4^+^ lymphoproliferation, lymphocytic tissue infiltrates, and elevated proinflammatory cytokine levels characterize the disease [73–75]. However, it was not until the early 2000s that the genetic defect, localized in the centromeric region of the X chromosome (Xp11.23-Xq13.3) was identified [71] and closed the pathophysiological loop between the clinical presentation of IPEX disease, the crucial role of FoxP3 signaling and regulatory T cells functions [4, 6, 76]. Since then, IPEX syndrome is also named according to the molecular defect as FoxP3 deficiency. Independent of the genetic mutation, the clinical presentation of patients with IPEX varies substantially. In a cohort of 96 IPEX patients, 39 had neonatal onset of enteropathy, type I diabetes, and eczema, and in less than half of the patients, nephropathy, autoimmune cytopenia, hepatitis, or thyroiditis. Only a few patients present with arthritis, alopecia, lymphadenopathy, or neutropenia [77]. Vice versa type I diabetes may be the only clinical sign at time of diagnosis [78, 79].

Meanwhile, numerous additional monogenetic diseases that lead to clinically relevant Treg deficiency have been described. These diseases share mutations in individual genes encoding proteins that crucially support regulatory T-cell functions. In the last update in 2022, the International Union of Immunological Societies (IUIS) classified these pathologies as *diseases of immune dysregulation*, summarized in the IUIS group IV. Most of these diseases result from monogenetic loss-of-function mutations (LOF), *e. g.* in *FOXP3, CD25, LRBA, CTLA4, AIRE, IL-10, IL-10R, STAT5B, or BACH2.* Additionally, gain-of-function mutations (GOF) in *STAT3*, may lead to a similar phenotype. However, as the genetic and phenotypic heterogeneity of Treg deficiencies makes the clinical diagnosis difficult, the term “*Tregopathies*” has been aptly established [80]. Laboratory findings in patients with numeric or functional Treg deficiencies are only indicative and require specific Treg staining because lymphocyte distribution frequently shows largely normal results. Furthermore, despite the shared clinical characteristics of genetically different Treg pathologies, understanding the underlying functional pathomechanisms to direct target-specific therapies is crucial.

### CTLA4-deficiency

As a member of the immunoglobulin superfamily and a costimulatory molecule that transmits inhibitory signals to T cells, CTLA-4 is a key element in Treg-mediated immune regulation [4, 81]. The phenotypic overlap between FoxP3, CTLA-4 and also TGF-β deficiency was first described in mice [82–85]. Tregs are severely impaired in patients with CTLA4 haploinsufficiency. The clinical phenotype is dominated by immune dysregulation with autoimmunity, immunodeficiency, and lymphoproliferation (IDAIL) but shows a highly variable presentation [86–90]. Based on its genotypic background, this syndrome is also referred to as CHAI (CTLA-4 Haploinsufficiency with Autoimmune Infiltration). Although late disease onset is possible, most patients report their initial clinical signs during early childhood. In a cohort of 133 patients from 54 unrelated families with genetically confirmed CTLA4 deficiency initial symptoms included autoimmune cytopenia (33%), respiratory manifestations (21%), enteropathy (17%), type 1 diabetes (8%), neurologic symptoms (seizures and headache (6%)), thyroid disease (5%), arthritis (3%), growth retardation, fever or night sweats, atopic dermatitis, or alopecia [87]. The pattern of lymphocytic organ infiltration was found to be heterogeneous, even among members of the same family [86–89]. Susceptibility to infection in CHAI reflects the characteristics of combined immunodeficiency with infections predominantly caused by *Hemophilus influenzae, pneumococci, Salmonella enteritidis, and* fungal species. Both EBV and CMV infections may affect multiple organs and typically recur [87]. While the frequency of circulating FoxP3 + Tregs may be normal or even increased, the expression of both CLTA-4 and FoxP3 is substantially reduced and must be specifically requested during immunological workup. Insufficient CTLA-4-mediated T-cell inhibition results in increased T-cell activation and the loss of naive CD45RA + CD4^+^ T cells. Dysregulated activation of the T-cell compartment contributes to autoimmune manifestations and uncontrolled lymphoproliferation. Without sufficient control of non-specific T-cell activation, pathogen-specific immune responses and lymphocytic maturation are compromised. Altered T-/B-cellular interactions may result in hypogammaglobulinemia and the formation of autoreactive antibodies [87, 88, 90]. Similar to Foxp3 deficiency, pathogenic mutations in *DEF6* may lead to a secondary CTLA-4 deficiency [91]. The guanine nucleotide exchange factor DEF6, also known as IRF-4 binding protein (IBP), interacts downstream of TCR with the GTPase RAB11 and is crucially involved not only in CTLA-4 availability and trafficking, but also in multiple processes of the innate and adaptive immune system, particularly T-cell differentiation, expansion, and maturation [91, 92].

### LRBA deficiency

CTLA4 recycling to the cell surface is further dependent on lipopolysaccharide-responsive and beige-like anchor protein LRBA. LRBA deficiency, which is typically associated with autoantibodies, regulatory T (Treg) cell defects, autoimmune infiltration, and enteropathy (called LATAIE) was first described in 2012 [93, 94]. Although the initial clinical manifestation within the first four years of life is dominated by autoimmunity including antibody-mediated cytopenia or endocrinopathy (42%), chronic diarrhea (27%) recurrent infections particularly of the respiratory tract (16%) and lymphoproliferation (5%), other signs of manifestation should not be missed [95]. The latter may include asthma and allergies, fever or unspecific failure to thrive. Later, autoimmunity (82%), enteropathy (63%), splenomegaly (57%) and pneumonia (49%) dominate the clinical course of disease [95],, but are accompanied by lymphadenopathy and lymphocytic tissue infiltration [96–99]. Homozygous mutations in the *LRBA* gene result in the loss of LRBA and impaired cellular signalling, dysfunctional vesicular trafficking, and lysosomal degradation of CTLA-4. Tregs in LRBA deficient patients are both numerically and functionally reduced with decreased expression of the most important Treg markers FoxP3, CD25 and CTLA4 [97]. The suppressive capacity of LRBA-deficient Tregs is significantly impaired, although IL-10 production seems to increase compared to healthy controls [97]. Uncontrolled T-cell activation leads to a loss of naivety and enhanced T-cell turnover. Dysregulated T follicular helper cells and defective B-cell activation result in peripheral B-cell lymphopenia and early-onset hypogammaglobulinemia, which seems to be the primary cause for recurrent respiratory tract infections [100]. At the same time functional Treg deficiency promotes the development of autoantibodies [97, 100].

### IL-2 signaling deficiencies

Signaling events within the IL-2 pathway crucially involve the IL-2 receptor CD25 and the transcription factors STAT3, STAT5B and FoxP3. CD25 deficiency due to homozygous mutations in the IL-2 receptor alpha chain (IL2RA) was first described in 1997 by Sharfe et al*.* in a child with increased susceptibility to viral, bacterial, and fungal infections, lymphoproliferation and chronic lung disease [101]. As CD25 is critically involved in global T-cell activation, CD25 deficiency not only results in reduced and functionally impaired Tregs and autoimmunity but also in profound T-cell proliferation deficits and nonspecific lymphoproliferation [101–104]. In the absence of functional CD25, IL-2-mediated Treg differentiation and maturation is hampered, leading to substantially reduced and dysfunctional Tregs and increased numbers of autoreactive T cells [104]. Although the clinical picture of CD25 deficiency varies with respect to the sites of manifestation and severity, the triad comprising immune dysregulation, autoimmunity, and severe susceptibility to viral, bacterial, and fungal infections has been described almost all patients [101–105]. The profoundness of this disorder is not only indicated by the manifestation severity but also by the early onset of the disease (mean 1.25 months) [106]. As the disease progresses, autoimmune-mediated cytopenia, hepatitis, pneumopathy and small vessel pulmonary vasculitis may occur [102, 105, 107]. Closely related, impaired IL-2 signalling may be caused by a homozygous mutation in the IL2RB (CD122) gene [108]. Affected individuals present with life-threatening immune dysregulation, including nonspecific but severe lymphoproliferation, enteropathy, eczema, and susceptibility to viral infections, particularly CMV, during early infancy [108, 109]. The immunological phenotype further includes impaired NK cell differentiation despite increased peripheral NK cell frequencies and combined immunodeficiency with hypergammaglobulinemia and autoantibody formation [108, 109].

### IL-10/IL-10R deficiencies

As IL-2 responsiveness is important for IL-10 induction in CD4^+^ T cells, patients with CD25 deficiency share severe and early onset inflammatory bowel disease with patients suffering from IL-10 or IL-10R (IL-10RA or IL-10-RB) deficiency [102, 110–112]. IL-10 is involved in the development and maintenance of Tregs and supports Treg-mediated suppression of pathogenic Th-17 cells in a STAT3 dependent manner [62, 113]. In in a cohort of 286 patients with IL-10/IL-10R deficiency, gastrointestinal disorders occurred in all patients with perianal manifestations (92%), fistulae (60%), and colitis (50%) being the most prominent signs, Interestingly, perianal abscesses (57%) and complications beyond the gastrointestinal tract including failure to thrive (58%), susceptibility to infections (≤ 23%), lymphoproliferation (≤ 12%), dermatologic manifestations (49%), or rheumatologic disorders (13%) were strictly linked to IL-10R deficiences and did not occur in IL-10 deficient patients [114].

### STAT3 and STAT5B signaling deficiencies

Downstream of the IL-2 and IL-10 receptor STAT3 acts as transcription factor that orchestrates multiple cellular functions. Furthermore, STAT3 acts as a key regulator in multiple signaling cascades downstream of *e. g.* receptors that involve the common gamma chain (IL-2, IL-4, IL-9, IL-15, IL-21) [115–119], receptors of the interferon family [120] and hormone receptors. Upon receptor activation STAT3 is phosphorylated in a JAK-dependent fashion and subsequently translocates to the nucleus where it binds as homo- or heterodimer to responsive elements triggering the transcription of cytokine responsive genes [121]. Loss of function mutations result in early-onset eczema, bacterial and fungal infections particularly of the skin and the lung, facial dysmorphism and joint hyperextensibility accompanies by elevated serum IgE levels [122]; but autoimmune phenomena are very rare [123]. In contrast, gain-of-function mutations in STAT3, which follow an autosomal inheritance, usually manifest during early childhood as a poly-autoimmune disease with lymphoproliferation, polyendocrinopathy, enteropathy, cytopenia, and severe interstitial lung disease. Further, increased susceptibility to infections, eczema, and short stature has been reported in these patients [124–127]. The functional details of enhanced STAT3 are only partially understood and the cellular phenotype in STAT3 GOF patients seems heterogeneous, but absolute T-cell and B-cell numbers are characteristically reduced, and frequently associated with hypogammaglobulinema and impaired antigen-specific B-cell maturation and hypogammaglobulinemia [124–126]. At the molecular level, activated STAT3 induces the expression of SOCS3, an inhibitor of STAT5 [128]. Accordingly, STAT3 GOF mutations result in secondary STAT5b deficiency [128]. As STAT5 itself is a crucial transcription factor for the expression of FOXP3, reduced FoxP3 expression can be observed in most patients with STAT3 GOF, and Tregs are functionally impaired [129–132]. This further explains why GOF mutations in STAT3 and LOF mutations in STAT5B share not only multiple aspects of the immunological IPEX-like phenotype including severe immune dysregulation but is also characterized by short stature due to (partial) growth hormone insensitivity [128, 133–137]. The clinical picture caused by pathogenic STAT5B mutations further includes severe pneumopathy, variable immunodeficiencies associated with susceptibility to severe sinopulmonary, dermal, and gastrointestinal infections. Initially described to follow an autosomal-recessive (AR) inheritance [134], STAT5B LOF mutations were identified to also occur as autosomal-dominant (AD) negative pathogenic variants in 2018 [137]. While growth retardation and eczema frequently occur in both AR and AD disease, additional clinical features occur not only in milder manifestations but also in less than 10% of patients with AD disease [106, 138]. The latter might explain why only few cases have been reported so far and suggests a high number of unrecognized cases. In summary, although defects in the IL-2 signalling pathway share many clinical features, depending on the defective molecule clinical phenomena differ in terms of the severity and frequency. For example, while eczema is with a prevalence of > 50% similarly frequent in patients with LOF mutations in *FOXP3*, *CD25*, *STAT5B* or GOF mutations in *STAT3,* autoimmune phenomena including cytopenia, thyroiditis and hepatitis as well as lymphoproliferations are characteristic for CD25 deficiency but occur in only less than 10% of patients with STAT5 deficiency [106, 129, 135, 139].

### CARMIL2 deficiency

Early onset skin lesions, including eczematous dermatitis associated with chronic mucocutaneous candidiasis, molluscum contagiosum bacterial abscesses, warts, inflammatory plaques, and hyperkeratosis, are characteristic hallmarks of CARMIL2 deficiency. Early reports on patients with homozygous CARMIL2 mutations further described a particular susceptibility to EBV infections, although recurrent and severe infections by other viruses, bacteria, mycobacteria, and fungi may occur [140–143]. Secondary to EBV infection, affected individuals show an increased risk of EBV-associated tumors [142, 144]. The *CARMIL2* gene is located on chromosome 16q22.1, and encodes the cytosolic protein CARMIL2 (Capping Protein Arp 2/3, Myosin-I Linker), also known as RLTPR [145], which acts as a scaffold bridging the CD28 to CARD11 and NFκB signaling cascades [146]. Functional analysis of CD4^+^ and CD8^+^ T-cell responses confirmed deficient CD3/CD28 costimulation in CARMIL2 deficient individuals [143]. While peripheral T-, B-, and NK cell counts are typically normal, Tregs are profoundly reduced. Due to deficient T-cell maturation, both CD4^+^ and CD8^+^ T-cell subsets are skewed towards naïve forms [140–143]. Within the B-cell compartment, class-switched B cells and plasmablasts may be reduced and show impaired immunoglobulin formation [141, 142]. Clinically, this results in a combined immunodeficiency syndrome with profound, early onset skin and inflammatory bowel disease and susceptibility to infections [147–149].

### BACH2 deficiency

The transcription factor BACH2, a highly conserved basic leucine zipper protein, is a key modulator of multiple immune processes, including T- and B-cell differentiation and maturation [150–152]. The *BACH2* locus contains a T-cell super-enhancer that regulates the expression of multiple pro-inflammatory cytokines and cytokine receptors [153, 154] and thereby reducing effector T-cell differentiation. In Tregs, BACH2 induces high FoxP3 expression, thereby promoting Treg development, maturation, and survival [151, 155]. BACH2 haploinsufficiency causes low Treg frequency and function, while differentiation of Th1-cells, which express the intestinal homing receptors CCR9 and ITGB7, is enhanced [97]. Similarly, the lack of BACH2 mediated repression of Th-2 differentiation results in increased Th-2 cytokine formation, promoting both airway and bowel inflammation [156]. As the effects of BACH2 deficiency manifest at every level of B-cell development, B-cell maturation and IgG class switch are profoundly impaired, resulting in increased transitional B-cell numbers, low immunoglobulins, and inability to generate appropriate antibody responses to specific antigens of vaccines. Accordingly, the clinical picture of BACH2-related immunodeficiency and autoimmunity (BRIDA syndrome) syndrome is dominated by sinopulmonary infections and autoimmune gastrointestinal diseases, which may present early in life [154].

### Tregs in autoimmune diseases

Unlike monogenetic Treg disorders polygenetic or multifactorial Treg deficiencies involve a complex interplay of multiple genes and environmental factors. Due to the multifactorial and polygenetic nature of these diseases understanding the interwoven factors contributing to the specific pathophysiology remains challenging. As mentioned earlier, we now know that Tregs represent a diverse subpopulation characterized by distinct transcriptional repertoires influenced by tissue- or context-specific transcription factors. For example, Tregs residing in adipose tissue express the transcription factor PPARγ, whereas those critical for driving Th1-type responses increase Tbet [58, 157, 158]. However, our current challenge is to use this knowledge to identify biomarkers that indicate Treg function in clinical settings. The broad spectrum of Treg functions makes the selection of a single marker or in vitro functional assay challenging, particularly in the context of a particular disease. The difficulties become even greater when assessing Treg activities in humans, primarily because of the obstacles associated with isolating Tregs from tissues other than blood.

### Tregs in type 1 diabetes

Type 1 diabetes (T1D) is the best-characterized autoimmune disease, and is often referred to as Juvenile Diabetes. It is a persistent autoimmune ailment characterized by a targeted immune response driven by both T- and B-cells, culminating in the destruction of insulin-producing β-cells nestled in the pancreatic islets [159]. T1D is one of the most common chronic metabolic diseases, affecting approximately 1.5 million people under 20 years of age [160]. It is one of the most frequent chronic metabolic diseases in childhood and adolescence, with a global increase in the incidence rate of 3–4% per year and strong regional differences [161]. Many autoimmune disorders, including T1D, frequently share disruptions in the control of effector cell populations as a fundamental contributing element [162, 163], and this aberration might stem from irregularities in the suppressive functions governed by Tregs.

A significant number of studies have indicated no disparities in peripheral blood Treg frequencies among T1D patients [164]. Nonetheless, anomalies in Treg phenotype and their suppressive potential have been documented [165, 166]. As described above, the challenge of obtaining healthy human tissue is particularly daunting when studying the role and function of Tregs in T1D, as pancreatic samples can only be obtained postmortem. Unfortunately, the unavailability of pancreatic samples from T1D patients has primarily confined data collection to peripheral blood, obscuring whether Tregs actively mitigate β-cell destruction or exhibit modified traits within islets during disease progression. Consequently, animal models such as mice have been harnessed to scrutinize disease advancement within the islet microenvironment.

Therefore, non-obese diabetic (NOD) mice are an essential model for T1D research. NOD mice spontaneously develop autoimmune diabetes, typically commencing at approximately 12 weeks in females, with the incidence increasing until approximately 25 weeks [167]. Male NOD mice experience delayed onset and progression of diabetes. The incidence is approximately 70% in females and 30% in males, a difference potentially rooted in gender-based variances in the gut microbiome and hormonal fluctuations [168]. Environmental factors, including housing conditions and diet, have been implicated in autoimmune diabetes onset [167]. Genomic investigations have identified susceptibility loci termed as insulin-dependent diabetes (IDD) loci in NOD mice. A plethora of over 40 IDD loci has been cataloged, with the major histocompatibility complex (MHC) exhibiting the most substantial link to T1D incidence [167, 169, 170]. Although the NOD mouse manifests several similarities with human T1D, some distinctions persist. Nevertheless, NOD mice have emerged as valuable tools for elucidating the role of Tregs in autoimmune diabetes [167].

Undoubtedly, genetic susceptibility constitutes a fundamental cornerstone in the evolution of T1D, with a significant proportion of susceptible single nucleotide polymorphisms (SNPs) being closely linked to immune-related genes, thereby underscoring immune dysregulation. Particularly noteworthy is the robust correlation observed with genes that have considerable influence over Treg function, most prominently IL2RA, IL-2, PTPN2, CTLA4, and IL-10 [171, 172]. However, translation of these SNPs into functional outcomes has only been achieved in a few studies. Additionally, because numerous pivotal genes serve both effector T cell and Treg functions, deciphering the relative impact of allelic variants on regulatory and effector T cells poses a formidable challenge. Several studies have reported SNP-associated impairments in Treg function, with a particular emphasis on IL-2 signaling [173–175]. These findings, coupled with analogous findings in NOD mice highlighting a deficiency in IL-2 signaling within Tregs, have galvanized efforts to harness this pathway for therapeutic intervention [175].

A regrettable adverse facet of low-dose IL-2 therapy, which effectively amplified Tregs, was the simultaneous escalation of eosinophils and natural killer cells, coupled with a reduction in C-peptide levels [176]. However, recent studies on Treg-specific IL-2 administration hold promise for overcoming off-target effects [177–179]. Furthermore, novel methodologies geared towards manipulating the pharmacokinetics of IL-2 therapy are expected to enhance its efficacy. One notable study employed the administration of low-dose IL-2/CD25 fusion protein, forestalling diabetes onset and even managing overt diabetes in the NOD mouse model of T1D. The augmented half-life of this IL-2 analog facilitates prolonged interaction with CD25-expressing Tregs, thereby amplifying IL-10 production and encouraging its migration to the pancreas [180].

More recently, a study harnessed T-cell population-specific epigenetic analysis to precisely locate susceptible SNPs within enhancer regions pivotal for Treg function in autoimmunity [181]. Comparative epigenetic evaluations across Treg and conventional T-cell populations revealed that autoimmune-associated SNPs were enriched in naïve Treg-specific demethylated regions and, to a lesser extent, in activated Treg-demethylated regions. These insights suggest that autoimmune-linked SNPs exert a more profound influence on thymus-derived Treg development and function than on aberrant activation of autoimmune effector T cells.

Several pathways critical for Treg development, function, and lineage stability are perturbed in T1D, potentially resulting in Treg dysfunction. Although studies employing Tregs derived from peripheral blood have provided evidence of altered Treg function in T1D patients [182, 183], the extent to which peripheral blood can accurately reflect Treg function at the tissue site remains ambiguous. Mouse models, particularly the NOD model, have provided invaluable insights into the mechanisms underlying the Treg suppression of islet autoimmunity. These investigations have illuminated the notion that certain deficiencies in Treg function are exacerbated at the tissue site, with Treg deficits not always conspicuous in in vitro assays [184, 185]. It is plausible that an amalgamation of chronic inflammatory mediators, anomalies in the IL-2 signaling pathway, and diminished TCR diversity, among other factors, converge within the pancreatic tissue, collectively weakening Treg function [184–187]. An optimal therapeutic strategy tailored to Tregs should be meticulously devised to address this combination of defects by stabilizing FOXP3 [188, 189].

### Tregs in autoimmune hepatitis

The incidence of autoimmune hepatitis (AIH) is similar. Due to the much smaller number of patients, the data were more uncertain. A pioneering genome-wide association study (GWAS) identified mainly genes of the HLA complex as risk factors, such as in T1D, but none were directly related to Tregs [190]. In relation to AIH, research has shown that Tregs may play a significant role in this context. A couple of studies from Vergani et al. have observed that patients with AIH may have reduced Treg numbers in the peripheral blood compared to healthy individuals [191–193]. The reduction in Tregs may contribute to an uncontrolled immune response against endogenous liver cells, ultimately leading to inflammation and liver damage. In particular, in pediatric AIH, decreased Treg, characterized as CD3^+^CD4^+^CD25^+^ numbers and impaired Treg function have been documented. Nonetheless, in a more recent study that included FOXP3 in Treg characterization (CD3^+^CD4^+^CD25^high^FOXP3^+^), the opposite was described as patients with AIH had increased Treg numbers in the blood [194]. This is consistent with the intrahepatic observation that the number of Tregs increases during active disease [195]. Notably, the same group also observed the opposite result in untreated pediatric patients with AIH [196]. In addition, the more pronounced effect of standard steroid therapy on decreasing Tregs over T effector cells was striking, leading to an increase in the apoptosis of Tregs [195, 197].

In mice, knockout of genes related to Treg development or function, such as aire or pd-1, leads to fatal autoimmunity in the liver and other organs, accompanied by decreased or absent numbers of Tregs [198–200]. In contrast, in animal models of AIH that resemble different aspects of human disease, an intrahepatic increase in Tregs in active AIH has also been observed [201–204].

### Tregs in colitis

The role of Tregs in colitis is closely linked to the balance between the inflammatory and regulatory immune responses in the gut. Studies have shown that Tregs play a key role in preventing excessive inflammatory processes in the gut. Patients with colitis, particularly Crohn's disease (CD) and ulcerative colitis, have been observed to have deficiencies in the number and function of Tregs [205–208]. This, in turn, promotes an unbridled and chronic inflammatory response that can damage intestinal tissue.

Previously conducted genome-wide association studies have identified over 100 separate genetic loci that contribute to either susceptibility or defense against the development of inflammatory bowel disease (IBD), with a considerable portion of these loci being shared between the two conditions [209, 210]. Administration of NOD2 ligands, including peptides or muramyl dipeptides, has demonstrated the potential to alleviate colitis induced by 2,4,6-trinitrobenzenesulfonic acid (TNBS) or dextran sulfate sodium (DSS) in normal mice [211, 212]. In a TNBS-induced colitis model, treatment with Lactobacillus peptidoglycan increased the number of Tregs in mesenteric lymph nodes and elevated IL-10 expression in the colonic mucosa, implying that NOD2 activity within the intestinal mucosa fosters a milieu conducive to immune tolerance. Furthermore, receptors associated with T cell and Treg migration, such as CD62L, C–C chemokine receptor (CCR)4, CCR5, CCR7, CCR9, αEβ7 integrin, and α4β7 integrin, also contribute to the pathogenesis of IBD [213–219]. The presence of these receptors on Treg cells plays a pivotal role in maintaining intestinal immunological equilibrium, and their compromised expression has been linked to the development of IBD, owing to the impaired migration of Treg cells into the intestinal tract. For example, the absence of CCR7 impedes Treg cell functionality in an experimental colitis model [214].

### Tregs as therapeutic agent or target

Among genetic and multifactorial autoimmune diseases, IBD is the most promising disease for polyclonal Treg transfer therapies. It has been shown, that Tr1 cells inhibit the proliferation of antigen-specific T cells through an IL-10-dependent mechanism and exhibit protective effects in the adoptive transfer model of colitis involving naïve T cells, as in SCID patients with colitis [220]. Although both FOXP3 + Treg cells and Tr1 cells generate IL-10, Tr1 cells appear to play a crucial role in upholding tolerance towards commensal bacteria. Nonetheless, Battaglia et al. demonstrated the necessity of FOXP3 + Treg cells next to Tr1 cells to persist for the initial induction of tolerance in autoinflammatory diseases [221–223]. We have highlighted the importance of Tregs to home to the gut and expand into the lamina propria to regain immunological tolerance [224]. As observed in SCID patients, the transfer of polyspecific Tregs is sufficient to treat autoimmune diseases that lack functional Tregs, such as SCID or APS-1 patients. Later, this was shown in other studies in the corresponding aire-deficient mouse model [199, 225–228]. To ensure the safety and efficacy of poly- or ovalbumin-specific autologous Tregs in the treatment of CD, many clinical trials have been conducted, including NCT03185000 (TRIBUTE) [229, 230], Eudract no. 2006–004712-44 [231], NCT02327221, NCT05566977, NCT03011021, NCT02932826, and NCT02691247. In addition, also antigen-specific Tregs bearing a chimeric antigen receptor (CAR) against model antigens were very successful in controlling colitis in animal models [232–235].

In T1D, the situation is very different, as polyspecific Tregs show no positive effects in mouse models or patients. It has been shown that antigen-specific Tregs are required to control diabetes and prevent its induction. Considering that Treg insufficiency potentially fuels T1D and autoimmune diabetes, bolstering the Treg count in circulation could serve as a strategy to counter this inadequacy. Notably, recurring adoptive Treg transfers into neonatal NOD mice have demonstrated the ability to postpone the onset of autoimmune diabetes, implying that Treg number or functionality might wane in NOD mice over time, necessitating supplementation [236]. Many T1D studies use BDC-2.5 mice, which are genetically modified NOD mice, carrying a transgenic TCR that recognizes a pancreatic antigen in NOD mice. These T cells destroy the insulin-producing cells in the pancreas. Therefore, another convincing strategy involves the adoptive transfer of a small quantity of DC-expanded BDC2.5 TCR-tg Tregs into pre-diabetic NOD mice. The transfer successfully prevented diabetes development and even salvaged mice with manifest diabetes [237]. When pre-diabetic NOD splenocytes or BDC2.5 TCR-tg Teff cells are transplanted into immunodeficient NOD mice, autoimmune diabetes typically emerges approximately 14 d post-transfer. Interestingly, co-transplantation with over a million polyclonal Tregs or a few thousand BDC2.5 TCR-tg Tregs can prevent the disease [238]. While a minimal number of antigen-specific Tregs have the capacity to reverse autoimmune diabetes, adopting ten-fold more polyclonal Tregs was not as effective in the therapeutic treatment of NOD mice, underscoring the critical significance of specificity for β-cell antigens in optimizing Treg functionality [239–243].

Clinically, in vitro-expanded polyclonal Tregs are being evaluated as a promising avenue that diverges from pharmacologically based treatments. Early phase clinical trials encompassing both pediatric and adult participants with autologous, polyspecific Tregs have been conducted, reflecting no immediate safety concerns, such as ISRCTN06128462, NCT01210664, NCT02932826, and NCT02772679 [244–247]. Notably, in children, potential efficacy has been assessed based on C-peptide levels at 4–5 weeks post-treatment. However, while initial elevations in C-peptide levels were evident at the one- and two-year follow-ups, they gradually diminished over time. Intriguingly, nearly 25% of transferred Tregs, characterized by a naïve/memory-like profile, persisted in patients at the one-year follow-up based on deuterium incorporation. A parallel trial conducted in Poland has yielded encouraging outcomes. In a cohort of 12 children with T1D, a one-year follow-up revealed augmented C-peptide levels and reduced insulin usage in 8 of 12 patients, resulting in complete insulin independence in 2 of the 12 patients [244–247]. Whether these encouraging observations endure and can be replicated in phase 2 clinical trials remains to be ascertained.

The potential for a more robust success may rely on combination therapy. Potential synergies with Tregs have been explored to optimize their therapeutic response in different autoimmune diseases. One effective strategy could involve bolstering the Treg population through the infusion of ex vivo expanded Tregs, while concurrently reducing the Teff population using agents such as anti-CD3 monoclonal antibody (NCT00129259) [248] or LFA3-Immunglobulin (Ig) (NCT00965458) [249, 250], which have shown promise in initial trials for new-onset T1D, followed by more than 30 other trials with similar results.

Another avenue to consider is the coupling of Treg cell infusion with interleukin-2 (IL-2). In vivo, IL-2 at low doses plays a pivotal role in the growth and survival of Tregs [251, 252], and constitute a critical component of Treg expansion protocols. Either Tregs might be directly equipped with IL-2 signals [253] or employing low-dose IL-2 has been effective in enhancing endogenous Tregs, leading to diabetes prevention and reversal in the NOD mouse model [184, 254]. Preliminary clinical investigations involving low-dose IL-2 have demonstrated selective increases in Tregs, yielding positive clinical outcomes in many (auto-)inflammatory conditions [255–259]. While a clinical trial combining IL-2 with rapamycin showed transient deterioration in beta cell function, potentially due to the relatively higher IL-2 dose or the influence of rapamycin, which led to significant increases in natural killer cells and eosinophils, early studies on lower IL-2 doses in T1D have shown no acute degradation in beta cell function [260, 261]. Several ongoing studies are aimed at evaluating the safety and efficacy of this approach. In an upcoming phase I trial involving autologous ex vivo expanded Tregs followed by low-dose IL-2, we will assess whether low-dose IL-2 enhances in vivo survival and functionality of infused Tregs.

Tregs administered in the initial clinical trials on T1D did not exhibit TCRs or CARs that specifically targeted diabetes antigens. There are several possible explanations for the selection of polyclonal Tregs for such an administration. The safety aspect was the most important, as all studies validated the safety of polyclonal cells, which is a primary consideration in devising a clinical research protocol. Thus, the effectiveness of polyspecific Tregs has been demonstrated in animal models, and data from animal studies involving exogenous IL-2, which augments Treg numbers, suggest the efficacy of polyclonal cells. Unlike other cellular processes, Treg-mediated suppression lacks antigen specificity; hence, polyclonal T cells should be capable of regulating cells that possess specificities for diabetes antigens.

Preclinical investigations have proposed that antigen-specific Tregs are more efficient in controlling autoimmune-mediated beta cell destruction than polyclonal Tregs [239–243]. Although target antigens for T1D have been identified, a significant challenge lies in isolating these less common cells from peripheral circulation and subsequently expanding them for clinical application [262]. Hence, it may be advantageous to generate antigen-specific de novo Tregs. Engineered Tregs featuring CARs have exhibited success in pre-clinical T1D [263, 264] and could potentially be viable for clinical deployment, albeit the specific antigen profile might need customization for each individual patient.

Polyspecific Tregs were also used in a clinical trials for AIH (NCT02704338) [265], Pemphigus Vulgaris NCT03239470, and Lupus Erythematosus NCT02428309 proving safety. However, polyspecific Treg transfer was not very effective in AIH mouse models [201, 203]. Therefore, we and others have successfully used optimization protocols for Tregs to determine their therapeutic responses in different AIH models [266–268]. Nonetheless, data on combination therapy or antigen-specific CAR-Tregs are lacking.

Notably, the significance of antigen-specific CAR-Treg cells in preventing genetic resilience against organ-specific autoimmunity and inhibiting autoimmune tissue damage has been well documented in various disease models, including multiple sclerosis [269] and Alzheimer’s disease [270].

## Conclusion

Numeric and functional Treg deficiencies may be due to monogenetic, inborn errors of immunity or occur by a more complex multifactorial processes. While congenital global Treg deficiencies result in polytopic diseases and usually affect multiple organ systems, genetically undefined autoimmune diseases, such as T1D or AIH, are characterized by the lack of particular antigen-specific Treg subsets but normal Tregs numbers. With the rapidly growing understanding of the genetic and functional Treg biology, new therapeutic approaches are currently being developed to overcome unmet medical needs in Treg diseases. The adoptive transfer of ex vivo expanded or even genetically modified Treg products is in the focus of upcoming clinical trials. The success of these approaches will depend on both overcoming technical and regulatory hurdles and ensuring product safety and efficacy in larger patient cohorts. Undoubtedly, the possibility to tailor highly personalized Treg products that address patient or disease specific needs has certainly opened a new dimension of target specific treatment approaches.

## Data Availability

Not applicable.

## Abbreviations

AIRE: Autoimmune Regulator
AIH: Autoimmune Hepatitis
APS-1: Autoimmune Polyendocrine Syndrome Type 1
BACH2: BTB Domain and CNC Homolog 2 (a transcription factor)
CAR: Chimeric Antigen Receptor
CCR: C–C Chemokine Receptor
CD: Crohn's Disease
CD25: IL-2 Receptor Alpha Chain (also known as IL2RA)
CTLA-4: Cytotoxic T-lymphocyte-associated Protein 4
CXCR3: Chemokine Receptor
DC: Dendritic Cell
EBV: Epstein-Barr Virus
FOXP3: Forkhead Box P3 (a transcription factor crucial for Treg development and function)
GITR: Glucocorticoid-Induced TNFR-Related Protein
GWAS: Genome-Wide Association Study
IDD: Insulin-dependent diabetes
Ig: Immunoglobulin
IDO: Indoleamine 2,3-Dioxygenase
IFN-γ: Interferon-gamma
IL-2: Interleukin-2
IL-10: Interleukin-10
IL-10R: IL-10 Receptor
IL2RA: IL-2 Receptor Alpha Chain
IL-35: Interleukin-35
IPEX Syndrome: X-linked Immune Dysregulation, Polyendocrinopathy, and Enteropathy
IUIS: International Union of Immunological Societies
mTECs: Medullary Thymic Epithelial Cells
NOD: Non-obese Diabetic (mouse model)
PD-1/PD-1L: Programmed Cell Death Protein 1/Programmed Cell Death 1 Ligand 1
SNPs: Single Nucleotide Polymorphisms
STAT3: Signal Transducer and Activator of Transcription 3
STAT5: Signal Transducer and Activator of Transcription 5
STAT5B: Signal Transducer and Activator of Transcription 5B
T1D: Type 1 Diabetes
TCR: T-Cell Receptor
Teff cells: Effector T cells
Tregs: Regulatory T cells
Tr1 cells: Type 1 regulatory T cells
TSA: Tissue-Specific Antigens
Tregopathies: Diseases Causing Regulatory T-cell Deficiencies
TNBS: 2,4,6-Trinitrobenzenesulfonic Acid
tTregs: Thymically derived Tregs
pTregs: Peripherally induced Tregs
